# Morphometric analysis of the coracoid process and glenoid width: a 3D-CT study

**DOI:** 10.1186/s13018-020-01600-1

**Published:** 2020-02-24

**Authors:** Yaofei Jia, Na He, Jiaxin Liu, Guangrui Zhang, Jianping Zhou, Ding Wu, Baomin Wei, Xiangdong Yun

**Affiliations:** 1 Department of Orthopedics, Lanzhou University Second Hospital, No. 82 Cuiyingmen, Chengguan District, Lanzhou, Gansu China; 2 Key Laboratory of Orthopedics of Gansu Province, No. 82 Cuiyingmen, Chengguan District, Lanzhou, 730030 Gansu China; 3 People’s Hospital of Changwu County, Xianyang, 713600 Shaanxi China

**Keywords:** Coracoid process, Glenoid width, 3D-CT, Morphometric parameters, Relationship

## Abstract

**Background:**

Data regarding the parameters of the coracoid process and glenoid width are insufficient, and information on gender, age, and ethnic differences in the parameters appear lacking in the Chinese population. This study aimed to investigate the morphometric parameters in the coracoid process and glenoid width.

**Methods:**

Using our institution’s electronic database, we selected 84 patients (55 males and 29 females) who underwent a shoulder computed tomography (CT) scan from January 2017 to May 2018 in this study. Mimics19.0 software was used for three-dimensional (3D) reconstruction of CT and to measure the morphometric parameters of the coracoid process and glenoid width. Subgroup analyses stratified by gender and age were conducted and the parameters were compared with previously published reports. All data were statistically analysed by SPSS23.0 Statistical Package.

**Results:**

A positive and significant relationship between the coracoid process and the glenoid width (*R* > 0.758, *P* < 0.01) was found. The midpoint width represents 52% (41–62%) of the glenoid width; the midpoint height, 40% (31–53%) of the glenoid width. Significant differences in all parameters between males and females were noted (*P* < 0.05). No significant differences among the age groups were observed (*P* > 0.05), whereas significant differences in almost all parameters between the ethnic groups were observed (*P* < 0.05).

**Conclusion:**

Our results could supplement the information in the shoulder joint database with morphometric parameters and provide a reference for theoretical research on coracoid osteotomy, which may in turn help surgeons in the evaluation of coracoid process transfer.

## Introduction

As the most mobile and unstable joint in the human body, the glenohumeral joint is the most commonly dislocated joint. Patients with a significant glenoid bone loss remain to have a high failure rate after Bankart repair [1–3]. Numerous investigations have reported on glenoid reconstruction procedures that utilise the coracoid process, iliac crest [4], or femoral head distal tibia autologous bone, as well as distal tibial allografts [5–8]. Coracoid transfer to the anterior glenoid (Latarjet procedure) is the most common reconstruction procedure [9–11].

The Latarjet procedure, which is also known as the coracoid process transfer, was first described in 1954 and used “bone block” of the coracoid for anteroinferior shoulder instability [12]. The coracoid increases the surface area of the glenoid, and the conjoint tendon and intact subscapularis provide the “sling effect” [13, 14], which plays an especially important role in mid- to end-range shoulder abduction [15]. Whether a coracoid graft (Latarjet procedure) could sufficiently restore glenoid bone loss remains to be clearly established. In 2002, Edwards et al. [16] reported that in patients with bone loss of > 33% of the glenoid width, the coracoid bone could be insufficient to reconstruct the glenoid. In 2005, Chen et al. [17] demonstrated that for glenoid bone defects > 20–33%, the Latarjet, Bristow, or other bone-grafting procedures could be used to reconstruct the glenoid. In 2011, Abboud et al. [18] reported that for glenoid bone loss > 21–50%, the Latarjet procedure could be employed to restore the glenoid.

In addition, reverse total shoulder arthroplasty (RTSA) has been successful in improving function and pain in patients with severe rotator cuff arthropathy and glenohumeral arthritis [19, 20]. Glenoid component loosening is the dominant cause of failure in RTSA [21]. Importantly, optimal glenoid component design and ability to reconstruct the normal glenohumeral anatomy contributes to improved stability of glenoid component fixation and fewer complications [22, 23]. Therefore, the accurate anatomic data of glenoid bone is crucial for new glenoid component design and necessary for the pre-operative planning of RTSA.

Previous investigations reported the bony dimensions of the coracoid process and glenoid width using computed tomography (CT) scan data; however, the number of studies that define the morphometric parameters of the coracoid process and glenoid width based on 3D-CT reconstruction in the Chinese population is limited. Thus, the purpose of this study was to evaluate the morphometric parameters in the coracoid process and glenoid width using three-dimensional computed tomography (3D-CT) reconstruction and to determine whether a significant difference in the coracoid process and glenoid width dimensions based on sex, age, and ethnicity exists.

## Materials and methods

### Study participants

Using the electronic database of the Lanzhou University Second Hospital, 124 patients who underwent CT scan of the shoulder from January 2017 to May 2018 were selected. Inclusion criteria were as follows: age 20–60 years old and Constant-Murley score > 90; all patients signed written informed consent. The exclusion criteria were as follows: previous scapular or humeral fracture, previous shoulder surgery, periarthritis of the shoulder, tumours around the shoulder, and shoulder joint instability. After applying the exclusion criteria, 84 patients (55 males and 29 females) were included in this study. The study protocol was performed in accordance with the Declaration of Helsinki and was approved by the institutional review board of the Lanzhou University Second Hospital.

### Parameters of the measurement

**Fig. 1:**
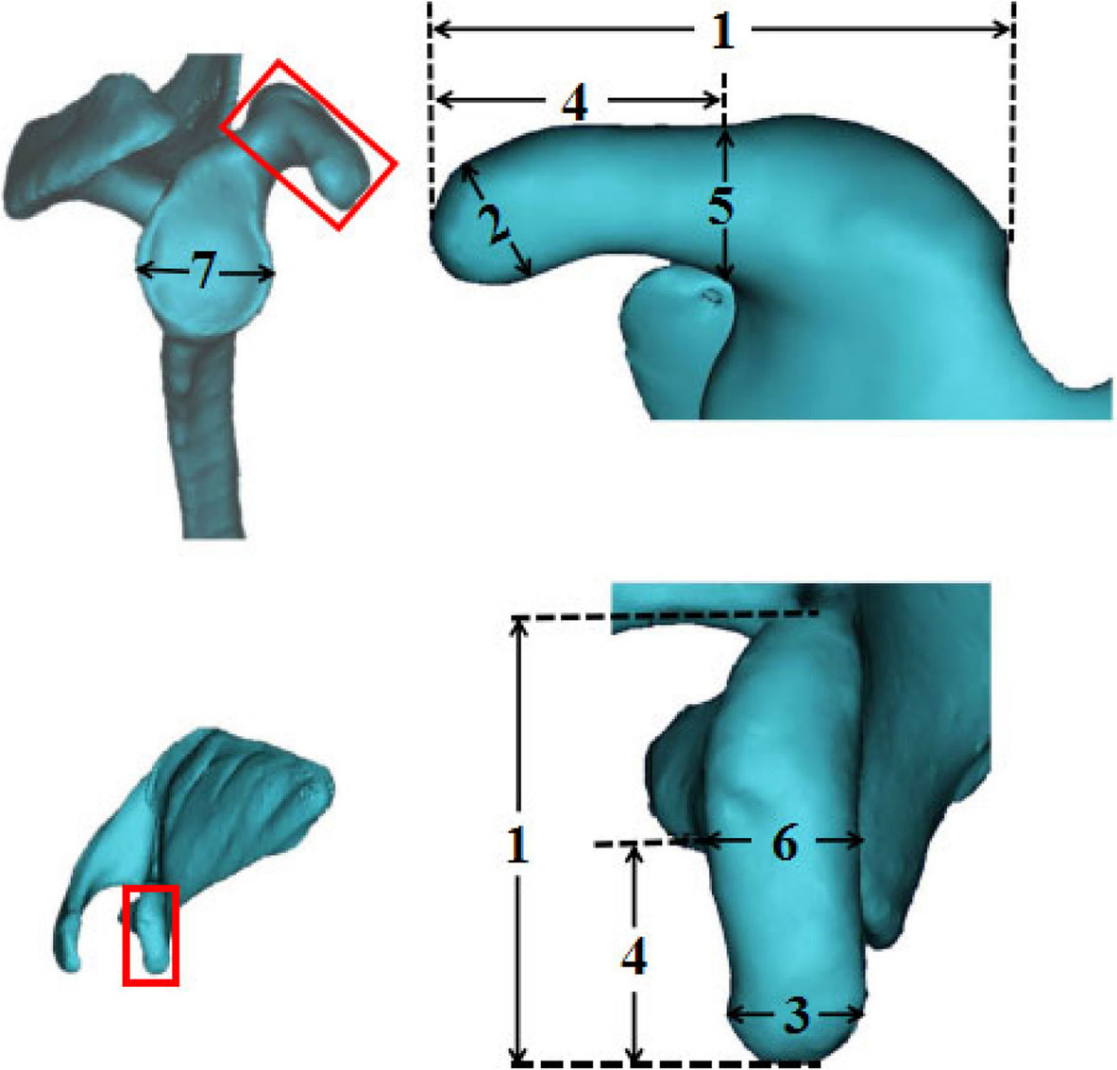
The coracoid process and glenoid cavity measurements. 1, coracoid length (distance from tip to base); 2, coracoid tip height; 3, coracoid tip width; 4, distance from the coracoid tip or base to the coracoid midpoint; 5, midpoint height; 6, midpoint width; 7, maximum anteroposterior diameter of the glenoid (glenoid width)

Mimics19.0 software (Materialise, Leuven, Belgium) was used to perform the 3D reconstruction of CT and to measure the anatomical parameters of the coracoid process and the glenoid width. All parameters were performed by two researchers who were blinded to each other’s data, and the data from the two researchers were averaged and recorded. The following bony dimensions of the coracoid process and the glenoid width were measured for each patient: coracoid length (distance from tip to base), coracoid tip height, coracoid tip width, distance from the coracoid tip or base to the coracoid midpoint (hereafter termed “midpoint”), midpoint height, midpoint width, and maximum anteroposterior diameter of the glenoid (glenoid width) (Fig. 1).

### Statistical analysis

All data were statistically analysed using SPSS version 23.0 (IBM Corporation, New York, NY, USA) and expressed as mean ± standard deviation (SD). The parameters were compared between males and females using independent samples *t* test. A comparison of all parameters among four age groups was analysed using one-way analysis of variance. Correlations among all parameters were evaluated by Pearson correlation analysis. All parameters were compared with previously published reports using one-sample *t* test. All statistical tests were two-tailed, and a *P* value < 0.05 was considered statistically significant.

## Results

The mean age of all patients was 41.63 (± 12.06) years. The patients were categorised by age as follows: 20–30, 31–40, 41–50, and 51–60 years old. The bony dimensions (Fig. 1) were measured. The anatomic dimensions of the coracoid process and glenoid width are presented in Table 1. The ratio between the midpoint width, the midpoint height, and the glenoid width was calculated. Results showed that the midpoint width represented 41–62% of the glenoid width (52% on average) and the midpoint height represented 31–53% of the glenoid width (40% on average).

**Table 1:** Anatomic dimensions of the coracoid process and glenoid width

Variable (mm)	Mean ± SD	Range (mm)	
		Min	Max
Coracoid length	41.60 ± 4.04	29.6	49.51
Tip height	9.05 ± 1.83	5.62	14.26
Tip width	13.09 ± 2.06	8.46	19.65
Tip/base to midpoint	20.80 ± 2.02	14.80	24.75
Midpoint height	11.12 ± 1.97	7.40	15.77
Midpoint width	14.59 ± 2.07	10.54	18.28
Glenoid width	27.86 ± 2.69	21.68	33.46

As shown in Table 2, differences in all parameters between males and females were significant (*P* < 0.05), with the male parameters being consistently greater. By contrast, no significant differences in all parameters among the four age groups were found (*P* > 0.05) (Table 3).

**Table 2:** Comparison of the coracoid process and glenoid width according to gender

Variable (mm)	Male (*n* = 55) Mean ± SD	Female (*n* = 29) Mean ± SD	*P* value*	95% CI
Coracoid length	43.56 ± 3.20	37.90 ± 2.65	0.000	4.27–7.04
Tip height	9.82 ± 1.55	7.58 ± 1.35	0.000	1.56–2.92
Tip width	13.97 ± 1.81	11.43 ± 1.39	0.000	1.77–3.30
Tip/base to midpoint	21.78 ± 1.60	18.95 ± 1.32	0.000	2.14–3.52
Midpoint height	12.03 ± 1.72	9.41 ± 1.08	0.000	2.00–3.23
Midpoint width	15.54 ± 1.73	12.79 ± 1.35	0.000	2.02–3.49
Glenoid width	29.09 ± 2.27	25.52 ± 1.72	0.000	2.61–4.53

*P < 0.05 indicates statistical significance

**Table 3:** Comparison of the coracoid process and glenoid width according to age

Variable (mm)	20–30 (*n* = 21)		31–40 (*n* = 14)		41–50 (*n* = 24)		51–60 (*n* = 25)		*F* value	*P* value
	Mean ± SD	95% CI	Mean ± SD	95% CI	Mean ± SD	95% CI	Mean ± SD	95% CI		
Coracoid length	41.96 ± 3.91	40.18–43.74	40.92 ± 4.40	38.38–43.46	41.33 ± 3.47	39.86–42.79	41.96 ± 4.60	40.06–43.86	0.281	0.839
Tip height	9.08 ± 1.72	8.30–9.86	8.52 ± 1.99	7.37–9.67	8.76 ± 1.39	8.17–9.35	9.60 ± 2.12	8.73–10.48	1.373	0.257
Tip width	12.90 ± 1.69	12.14–13.67	12.99 ± 2.38	11.61–14.36	12.75 ± 1.88	11.95–13.54	13.64 ± 2.32	12.69–14.60	0.886	0.452
Tip/base to midpoint	20.98 ± 1.95	20.09–21.87	20.46 ± 2.20	19.19–21.73	20.67 ± 1.74	19.93–21.40	20.98 ± 2.30	20.03–21.93	0.281	0.839
Midpoint height	11.64 ± 2.22	10.63–12.65	10.55 ± 1.89	9.46–11.64	10.89 ± 1.62	10.21–11.58	11.23 ± 2.10	10.36–12.10	1.002	0.396
Midpoint width	14.40 ± 2.03	13.48–15.33	14.15 ± 2.14	12.91–15.39	14.52 ± 1.99	13.67–15.36	15.07 ± 2.17	14.17–15.96	0.717	0.545
Glenoid width	27.67 ± 2.90	26.35–29.00	27.30 ± 2.43	25.90–28.71	27.82 ± 2.52	26.75–28.88	28.36 ± 2.88	27.17–29.55	0.516	0.672

**Fig. 2:**
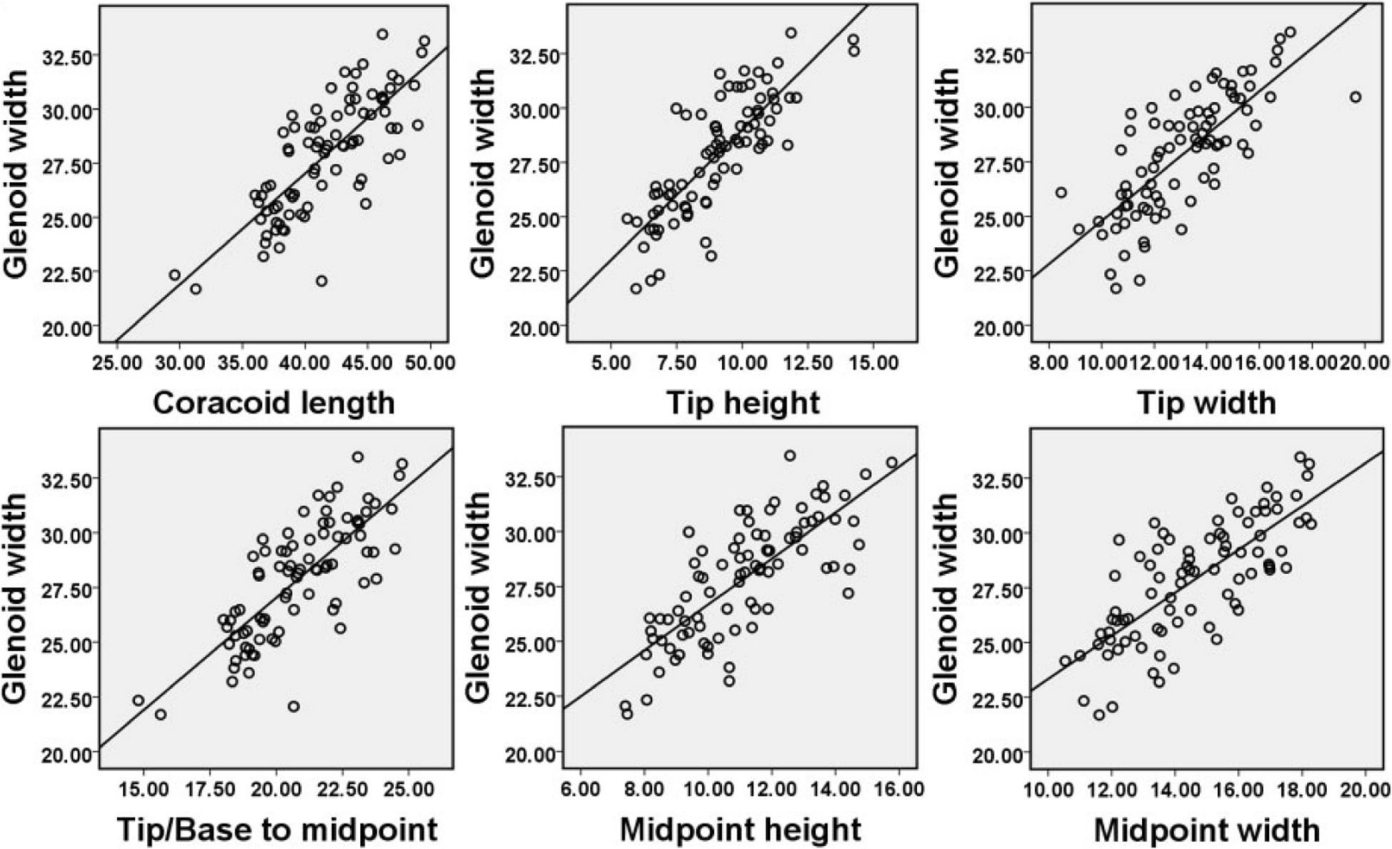
Correlation analysis between the coracoid process and the glenoid cavity dimensions

The Pearson correlation analysis and scatter plots are presented in Table 4 and Fig. 2. Data analysis revealed a significant positive correlation between the coracoid process and the glenoid width (*R* > 0.758, *P* < 0.01), for example, we observed a positive (*R* = 0.758) and statistically significant (*P* < 0.01) correlation between the midpoint width and the glenoid width, and a positive (*R* = 0.766) and statistically significant (*P* < 0.01) correlation between the midpoint height and the glenoid width was also noted.

**Table 4:** Pearson correlation analysis between the dimensions of the coracoid process and glenoid width

	Coracoid length	Tip height	Tip width	Tip/base to midpoint	Midpoint height	Midpoint width	Glenoid width
Coracoid length	1	0.737^**^	0.677^**^	1.000^**^	0.645^**^	0.707^**^	0.772^**^
Tip height	0.737^**^	1	0.820^**^	0.737^**^	0.813^**^	0.788^**^	0.813^**^
Tip width	0.677^**^	0.820^**^	1	0.677^**^	0.746^**^	0.846^**^	0.758^**^
Tip/base to midpoint	1.000^**^	0.737^**^	0.677^**^	1	0.645^**^	0.707^**^	0.772^**^
Midpoint height	0.645^**^	0.813^**^	0.746^**^	0.645^**^	1	0.750^**^	0.766^**^
Midpoint width	0.707^**^	0.788^**^	0.846^**^	0.707^**^	0.750^**^	1	0.758^**^
Glenoid width	0.772^**^	0.813^**^	0.758^**^	0.772^**^	0.766^**^	0.758^**^	1

**P < 0.01 indicates statistical significance

Furthermore, all parameters between the Chinese population and the populations from various countries and ethnic groups were compared (Table 5). Generally, Asians have shorter dimensions of the coracoid process and glenoid width than Caucasians and Africans (*P* < 0.05). Moreover, the coracoid length (37.94 ± 4.30 mm) and tip width (11.63 ± 2.12 mm) in the Malaysian population were found to be the smallest (*P* < 0.05). The distance from the coracoid tip/base to the midpoint was greater in the Mongolian Chinese (24.75 ± 7.23 mm) than in the American population (22.8 ± 2.1 mm).

**Table 5:** Measurements of coracoid process and glenoid width in different ethnic populations

Populations	Coracoid length (mm)	Tip height (mm)	Tip width (mm)	Tip/base to midpoint (mm)	Midpoint height (mm)	Midpoint width (mm)	Glenoid width (mm)	References
Asians								
Chinese	41.60 ± 4.04	9.05 ± 1.83	13.09 ± 2.06	20.80 ± 2.02	11.12 ± 1.97	14.59 ± 2.07	27.86 ± 2.69	Present study
Chinese	42.47 ± 1.02	9.08 ± 0.58	13.17 ± 0.51	–	–	–	–	[24]
Chinese	42.10 ± 2.31	9.10 ± 1.75	13.61 ± 2.00*	24.75 ± 7.23*	11.61 ± 1.98*	15.29 ± 1.70*	–	[25]
Indian	43.32 ± 1.54*	11.47 ± 0.62*	13.63 ± 1.09*	–	–	–	–	[24]
Indian	40.45 ± 4.43	–	–	–	8.54 ± 1.7*	14.16 ± 2.39	23.96 ± 3.22*	[26]
Myanmarese	39.19 ± 1.38*	8.58 ± 1.03	13.02 ± 1.32	–	–		–	[24]
Malaysian	37.94 ± 4.30*	9.24 ± 1.16	11.63 ± 2.12*	–	–	13.84 ± 1.76*	–	[27]
Caucasians								
American	46.0 ± 3.7*	9.2 ± 1.2	15.9 ± 1.9*	–	–	–	29.2 ± 2.5*	[28]
American	45.8 ± 4.2*	–	–	–	–	–	–	[29]
American	-	10.4 ± 1.4*	15.9 ± 2.2*	–	–	–	–	[30]
American	45.0 ± 3.8*	–	–	–	–	–	–	[31]
American	45.6 ± 4.2*	11.5 ± 0.9*	18.3 ± 1.8*	22.8 ± 2.1*	13.5 ± 1.6*	16.1 ± 2.3*	–	[32]
German	43.1 ± 2.2*	8.2 ± 1*	13.6 ± 2.1*	–	–	–	–	[33]
Canadian	–	10.5 ± 1.7*	15.0 ± 2.2*	–	–	–	–	[34]
Brazilian	42.6 ± 2.6	14.9 ± 1.2*	21.1 ± 2.0*	–	–	–	–	[35]
Brazilian	–	–	–	–	8.37 ± 0.93*	14.51 ± 1.90	26.38 ± 2.69*	[36]
Switzerland	–	–	–	–	–	–	27.8 ± 3.1	[37]
Africans								
African-American	44.4 ± 4.2*	9.4 ± 1.4	15.3 ± 2.1	–	–	–	28.1 ± 3.0	[28]
African-American	43.7 ± 3.8*	–	–	–	–	–		[29]

*P < 0.05 indicates statistical significance; – Represents no parameters in the study

## Discussion

Morphometric evaluation of the coracoid process, which is a potential mediator and key structure of shoulder pathology and surgery, has been performed by various authors in different populations using dry osteology, fresh cadaver, or 3D-CT reconstruction. However, data on the evaluation of the coracoid process morphology using CT scans in the Chinese population are extremely few. Moreover, data regarding the relationship between the coracoid process and the glenoid width are insufficient, and information on sex, age, and ethnic differences in the morphometry of the coracoid process appears lacking.

### The relationship between the coracoid process and the glenoid width

In our study, a positive and significant relationship between the coracoid process and the glenoid width was found (*R* > 0.758, *P* < 0.01), which is consistent with a previous study in an Indian population (*R* > 0.631) [26]. The average midpoint width/glenoid width ratio was 52% (range 41–62%), and the average midpoint height/glenoid width ratio was 40% (range 31–53%). This finding could be helpful for surgeons in restoring glenoid bone loss with a coracoid bone. In 2012, one study [36] reported that the midpoint width/glenoid width ratio represented 54% (43–70%) and the midpoint height/glenoid width ratio represented 31% (25–37%) of the glenoid width; moreover, a significant positive correlation between the coracoid tip width and the glenoid width was found (*R* = 0.677, *P* < 0.01), thereby strengthening the validity of the ratio in our results. In 2002, Edwards et al. [16] reported that in patients with bone loss of > 33% of the glenoid width, the coracoid bone could be insufficient for glenoid reconstruction. In addition, some authors recommended removing the coracoid cortical surface to increase the healing rate of the glenoid neck. Consequently, glenoid reconstruction was possible in most people with glenoid bone loss in our study.

### Gender differences in morphometric measurements of the coracoid process and the glenoid width

The coracoid morphological parameters stratified by gender showed that all parameters were significantly greater in males than in females, which is in agreement with the findings of previous studies [24, 29, 31, 33, 38]. Conversely, Imma et al. [27] found significant sex-related differences in the measurements except for the base height, tip length, and midpoint width. This conflicting result could be because the study was not stratified by ethnicities; moreover, the discrepancy could also be explained by two possible reasons: small sample size (15 pairs of shoulder joints) and a mixture of three ethnicities (Malay, Chinese, and Indian subjects).

### Age differences in morphometric measurements of the coracoid process and the glenoid width

To the best of our knowledge, only one study [28] reported on the morphology of the coracoid process and glenoid width based on age, which demonstrated significant differences in all measurements according to age (*P* < 0.001). The discrepancy between the previous study’s findings and our results could be attributed to the mixture of two ethnicities (i.e. Caucasians and African-Americans) in the former, and the measurements of the specimens vary (i.e. dry osteology vs. CT scan). Specifically, to eliminate the influence of confounding factors with increasing age, we excluded patients with the previous scapular or humeral fracture, previous shoulder surgery, periarthritis of the shoulder, tumours around the shoulder, and shoulder joint instability. Moreover, all measurements were performed by two researchers who were blinded to patients’ age, thereby minimising the potential observer bias.

### Ethnic differences in morphometric measurements of the coracoid process and the glenoid width

Parameters of the coracoid process and glenoid width in previously published reports vary, and a comparison of our parameters with those of previous studies is difficult as the studies have different ways of measuring the specimens (i.e. using dry bone, fresh cadaveric bone, or CT scan). For example, some previous studies defined the coracoid tip length as the distance from the coracoid tip to the “elbow” or “knee” of the coracoid [30, 34, 38]; therefore, the coracoid tip length was excluded in the comparison between the coracoid process and glenoid width among different ethnic populations. Generally, Asians have smaller coracoid process and glenoid width dimensions than Caucasians and Africans (*P* < 0.05), except for the distance from the coracoid tip/base to the coracoid midpoint among Mongolian Chinese [25]; the difference could be attributed to genetic as well as environmental influence, dietary habits, and lifestyle. Interestingly, no significant difference exists about glenoid width between the present study and a previous study [37] in Switzerland cadavers, which could be explained by the study focusing on body donors with an average age of 84 years (range 60–98 years), leading to degree of glenoid wear, and small sample sizes (18 cadavers).

This study has some inherent limitations. Because of the tortuous morphology on CT scans, identifying the bony landmarks was difficult. Although the measurements were performed by two researchers blinded to each other’s data, some errors were inevitable. Moreover, a previous study [25] reported that taller patients have a shorter coracoid height, and performing the congruent-arc Latarjet for these patients appears not a reasonable option as there is not enough space for the use of two 3.5-mm screws. However, we did not analyse the patient’s height. Thus, further investigations should include the evaluation of the patient’s height when performing the Latarjet technique in patients with glenoid bone loss.

## Conclusions

Our study demonstrated a positive and significant relationship between the coracoid process and the glenoid width, and a significant difference in the coracoid process and glenoid width dimensions based on sex and ethnicity was identified. These findings could supplement the data in the shoulder joint database with the anatomical parameters of the Chinese population, which may in turn help surgeons in evaluating the need for coracoid osteotomy and transfer during preoperative planning and intraoperative decision-making. Nevertheless, further studies with a larger sample size are required to validate our findings.

## Data Availability

Upon request, raw data can be provided.

## Abbreviations

3D: Three-dimensional
CT: Computed tomography
RTSA: Reverse total shoulder arthroplasty
SD: Standard deviation
